# 29791 Bird-delivered ivermectin as a novel urban West Nile virus prevention strategy

**DOI:** 10.1017/cts.2021.733

**Published:** 2021-03-30

**Authors:** Karen M Holcomb, Chilinh Nguyen, Nicholas Komar, Brian D Foy, Christopher M Barker

**Affiliations:** 1 University of California Davis; 2 Colorado State University; 3 Centers for Disease Control and Prevention Division of Vector-Borne Infectious Diseases

## Abstract

ABSTRACT IMPACT: Successful implementation of this control strategy will result in a commercially available ivermectin-treated birdfeed that the public can use to protect themselves from infection with West Nile virus (WNV) by reducing mosquito survival and thereby suppressing WNV transmission around their homes. OBJECTIVES/GOALS: We assessed the efficacy and feasibility of ivermectin (IVM)-treated birds as a mosquito control strategy for local reduction of West Nile virus (WNV) transmission. We conducted a randomized field trial in backyard chickens and developed a mathematical model informed by field data to predict the impact of treated wild birds on transmission. METHODS/STUDY POPULATION: We placed 48 chickens in four treated and four untreated control flocks in backyards coops across Davis, CA and administered IVM daily in feed to treated flocks (Jul-Sep 2019). We assessed entomological indices weekly (i.e. Culex mosquito abundance, WNV infection prevalence, and parity rate) around each coop, monitored serum IVM levels in treated chickens, and tested for WNV antibodies in all chickens. Shifting our focus to wild birds, we developed a spatially-implicit mathematical model of WNV transmission near IVM-treated birdfeeders. Model parameters for bird movement were based on our telemetry of 27 birds in Fort Collins, CO (Aug-Sep 2020). Using the model, we predicted optimal deployment of treated feeders to provide local WNV control. RESULTS/ANTICIPATED RESULTS: WNV seroconversions were reduced in treated vs. untreated flocks, indicating a reduction in WNV transmission intensity at treated coops (P = 0.03). A sustained, but insignificant reduction in number of infected mosquitoes was observed near treated coops (P = 0.59); small sample sizes and below normal WNV prevalence in the study area limited our power. We anticipate that optimal spacing and number of IVM-treated birdfeeders required for effective WNV control in neighborhoods will depend on feeder usage rates by common bird species irrespective of WNV competence; broad availability of IVM-treated bloodmeals to mosquitoes will be more effective in reducing transmission than targeting the few species responsible for viral amplification. DISCUSSION/SIGNIFICANCE OF FINDINGS: IVM is a novel method for controlling zoonotic pathogens in the US and has the potential for targeted mosquito control to reduce pesticide usage. Evaluating spatial deployment of IVM-treated bird feed for local reduction in WNV transmission is a stepping stone to commercial deployment of this WNV control strategy.

